# Association between smoking and chronic kidney disease: a case control study

**DOI:** 10.1186/1471-2458-10-731

**Published:** 2010-11-25

**Authors:** Rabi Yacoub, Habib Habib, Ayham Lahdo, Radwan Al Ali, Leon Varjabedian, George Atalla, Nader Kassis Akl, Saleem Aldakheel, Saeed Alahdab, Sami Albitar

**Affiliations:** 1 Internal Medicine department, University at Buffalo, Grider Street, Buffalo, N.Y. 14215, USA; 2 Internal Medicine department, Seton Hall University, Pennington Harbourton Road, Pennington, N.J. 08534, USA; 3 Internal Medicine department, John H. Stroger, Jr. Hospital of Cook County, West Ogden Avenue, Chicago, IL. 60612, USA; 4 Internal Medicine department, University of Aleppo, Almoohafaza Street, Aleppo. Syria

## Abstract

**Background:**

The progression of chronic kidney disease (CKD) remains one of the main challenges in clinical nephrology. Therefore, identifying the pathophysiological mechanisms and the independent preventable risk factors helps in decreasing the number of patients suffering end stage renal disease and slowing its progression.

**Methods:**

Smoking data was analyzed in patients with CKD throughout 2005-2009. One hundred and ninety-eight patients who had recently been diagnosed with stage three CKD or higher according to the National Kidney Foundation (NKF) 2002 Classification were studied. The control group was randomly selected and then matched with the case subjects using a computerized randomization technique. The relative risk was estimated by computing odds ratio (OR) by using multinomial logistic regression in SPSS ® for Windows between the two groups.

**Results:**

Smoking significantly increases the risk of CKD (OR = 1.6, *p *= 0.009, 95% CI = 1.12-2.29). When compared to nonsmokers, current smokers have an increased risk of having CKD (OR = 1.63 *p *= 0.02, 95% CI = 1.08-2.45), while former smokers did not have a statistically significant difference. The risk increased with high cumulative quantity (OR among smokers with > 30 pack-years was 2.6, *p *= 0.00, 95% CI = 1.53-4.41). Smoking increased the risk of CKD the most for those classified as hypertensive nephropathy (OR = 2.85, *p *= 0.01, 95% CI = 1.27-6.39) and diabetic nephropathy (2.24, *p *= 0.005, 95% CI = 1.27-3.96). No statistically significant difference in risk was found for glomerulonephritis patients or any other causes.

**Conclusion:**

This study suggests that heavy cigarette smoking increases the risk of CKD overall and particularly for CKD classified as hypertensive nephropathy and diabetic nephropathy.

## Background

Smoking, a well known risk factor for many diseases, was recently proven to play an important role in renal diseases. Studies showed that cigarette smoking is a risk factor for the development and progression of chronic kidney disease (CKD) in community [1,2]. In these studies, causes of CKD were heterogeneous, while other studies implied that the relationship between cigarette smoking and kidney impairment varied among underlying kidney diseases [3]. However, there is still uncertainty whether every kidney disease is equally vulnerable due to cigarette smoking. In this sense, further research is required.

Since urinary albumin is a sensitive marker of glomerular injury [4], it is conceivable that the relationship of smoking to albuminuria indicates direct or indirect renal damage induced by smoking. It is blamed for the deterioration of kidney function by increasing the risk of microalbuninuria [5], accelerating the progression from microalbuminuria to proteinuria [6,7] and as a result to diabetic nephropathy which leads to end stage renal disease (ESRD) [6,8]. In a prospective study with 794 patients who had non insulin dependent diabetes mellitus (NIDDM), who had no proteinuria at baseline, the relative risk of developing gross proteinuria (> 300 mg/day) during four years of observation was 2- to 2.5-fold higher in heavy smokers when compared to subjects who had never smoked [9]. Many studies also indicate a relationship between smoking and renal function deterioration in lupus patients, polycystic kidney disease, Goodpasture, renal artery stenosis, glomerulonephritis (GN) [10-12] and proximal tubular dysfunction [13,14].

This study aims to investigate the relationship between cigarette smoking and chronic kidney disease, and its effects on each type of renal failure.

## Methods

### Study Subjects and Data Collection

A cross-sectional study of 198 patients with CKD and 371 healthy control subjects were matched and studied. Cases were patients admitted or referred to one of the three tertiary hospitals affiliated with Aleppo university during 2005-2009 and newly diagnosed with CKD with estimated glomerular filtration rate (eGFR) of less than 60 mg/minute/1.73 m^2 ^(CKD stages 3-5, according to the National Kidney Foundation (NKF) 2002 classification) [15]. Patients with pre- or post-renal causes of CKD were not included in the study. The type of renal disease was determined by medical history, urinalysis, and renal biopsy. Eligibility and final diagnosis was confirmed by a university nephrologist who supervised the study. Case participants were enrolled after being approved and all were recently diagnosed with CKD. Control participants were randomly selected and then matched in gender and age from healthy people, using a computerized randomization technique based on Aleppo city national database. Eligible control subjects were people from the community who had no medical history of kidney disease, which was confirmed by normal eGFR reading (more than 90 mg/minute/1.73 m^2^) and a urine protein creatinine ratio less than 0.15 [16]. A registered research coordinator sent invitations through the mail. All measurements were done after the participants provided verbal or written consent and answered a questionnaire which included questions regarding demographic information and history of their renal disease and smoking habit.

The U.S. National Library of Medicine, Medical Subject Headings (MeSH) define smoking as inhaling and exhaling the smoke of tobacco or something similar to tobacco available at http://www.who.int. To reduce the probability that symptoms of early CKD are influenced by tobacco use, the classification of former versus current tobacco use was based on smoking status 5 years before the interview. Regular cigarette smoking was defined as smoking at least one cigarette per day for six months or more. Regular smokers were classified into either current regular smokers if they smoked during the last five years or former regular smokers if they quit more than five years ago. Tobacco consumption was measured with a pack per year formula.

### Statistical Analyses

As an estimate of the relative risk for CKD among tobacco users compared with non-smokers, we computed odds ratios (OR) in terms of SPSS^® ^(IBM^®^, Chicago IL. USA) for Microsoft Windows^®^, using multinomial logistic regression. We studied smoking as a relative risk for CKD, and then we studied former versus current smoking for the same purpose. The next step was to determine the relative risk of smoking for each type of renal disease. We studied analgesic (aspirin or paracetamol) use, body mass index (BMI), angiotensin-converting enzyme inhibitors (ACEi) or angiotensin II receptor blockers (ARBs) use, and hypertension (HTN) as confounding factors; however these factors were excluded from the final model because their inclusion did not affect the risk estimates.

## Results

One hundred and ninety-eight patients and 371 healthy participants as the control group were included. The most used tobacco form was as a cigarette, so we ignored pipe and snuff usage.

Fifty one percent of case subjects and 49% of control subjects were men (Table 1). The mean age of both groups was 45.36 ± 15.95 years, the case subjects' mean age was 45.15 ± 15.85 years and the control subjects mean age was 45.47 ± 16.00 years.

**Table 1:** Characteristic distribution in the case and control subjects

	Cases (n = 198)		Control (n = 371)		P value
			
	n	%*	n	%**	
Gender					0.57
Male	102	51.5	182	49	
Female	96	48.5	189	51	
Age (yr)					0.96
18-24	30	15.2	59	15.9	
25-34	28	14.1	48	12.9	
35-44	34	17.2	55	14.8	
45-54	44	22.2	92	24.7	
55-64	40	20.2	74	19.9	
≥65	22	11.1	43	11.5	
Medications					
Any HTN medication	91	45.9	149	40.1	0.18
ACEi	83	41.9	127	34.2	0.07
ARBs	15	7.6	26	7	0.81
Beta blockers	72	36.4	105	28.3	0.04
Analgesic	40	20.2	70	18.9	0.70
BMI					0.14
Underweight	7	3.5	26	7	
Normal weight	104	52.5	163	43.9	
Overweight	56	28.3	115	30.9	
Obesity	31	15.6	67	18	
HTN	58	29.3	92	24.8	0.25
Diabetes Mellitus	56	28.3	99	26.7	0.68

*Of cases (n = 198). **Of control (n = 371).

A majority of the patients were in The National Kidney Foundation Kidney Disease Outcomes Quality Initiative (NKF KDOQI ™ stage 3 and 4 [15]. Seventy percent had a creatinine level > 2 mg/dl. The median overall value of the eGFR calculated by the Modification of Diet in Renal Disease (MDRD) formula was 41 ml/min/1.73 m^2^. Forty five and a half percent of cases had glomerulonephritis as a cause for their CKD, 28.3% diabetic nephropathy, 13.1% hypertensive nephropathy, and 13.1% had an unknown or other causes for CKD.

About 43% of case subjects had ever smoked cigarettes regularly compared to 32% of control subjects. Compared to never regularly smoking, former and current regular smoking significantly increased the risk of CKD (OR = 1.6, CI 95% 1.12-2.29, *p *= 0.009) and (OR = 1.63, CI 95% 1.08-2.45, *p *= 0.02), respectively. The risk of CKD did not reach a significant level for former regular smokers (OR = 1.04, CI 95% 0.58-1.86, *p *= 0.8). This risk was obviously increased with the tobacco consumption, with OR = 2.6, CI 95% 1.53-4.41, *p *= 0.00 for more than 30 pack/year, and OR = 2.04, CI 95% 1.08-3.88, *p *= 0.02 for 16-30 pack/year compared to those who had never regularly smoked (Table 2).

**Table 2:** Smoking status and Odds ratio for chronic renal failure

	Cases		Control		OR^† ^(CI 95%)	P
				
	n	%*	n	%**		
Ever regular smoking						
No	112	56.5	251	67.7	1 (Reference)	-
Yes	86	43.4	120	32.3	1.6 (1.12-2.29)	0.009
Regular smoking						
Former	30	15.1	43	11.6	1.04(0.58-1.86)	0.8
Current	56	28.2	77	20.8	1.63(1.08-2.45)	0.02
No. of pack/years, cigarettes						
1-15	34	17.1	60	16.1	2.1(0.96-4.57)	0.06
16-30	16	8	29	7.8	2.04(1.08-3.88)	0.028
> 30	36	18.1	31	8.3	2.6(1.53-4.41)	0.000

*Of cases (n = 198). **Of control (n = 371).^† ^Adjusted by age and gender.

Table 3 presents OR for different types of CKD. Smoking was significantly associated with risk of CKD caused by hypertension (OR = 2.85, CI 95% 1.27-6.39, *p *= 0.01) and diabetic nephropathy (OR = 2.24, CI 95% 1.27-3.96, *p *= 0.005). This association did not reach a significant level for risk of CKD caused by glomerulonephritis (OR = 1.09, CI 95% 0.67-1.78, *p *= 0.7) and the other causes of CKD (OR = 1.53, CI 95% 0.68-3.44, *p *= 0.2). The control subjects were gathered from the same socioeconomic background. We did not find any influence by body mass index, hypertension and analgesic use on the final results.

**Table 3:** Odds ratio for chronic renal failure by type among regular smokers

	N (%*)	Ever regular use of cigarettes		OR^† ^(CI 95%)	P
				
		No n (%**)	Yes n (%+)		
Control	371(65.2)	120(51.7)	251(74.5)	1 Reference	-
Glomerulonephritis	90 (15.8)	59(25.4)	31(9.1)	1.09(0.67-1.78)	0.7
Diabetic nephropathy	56 (9.8)	27(11.6)	29(8.6)	2.24(1.27-3.96)	0.005
Hypertension	26 (4.6)	11(4.8)	15(4.5)	2.85(1.27-6.39)	0.011
Unknown and other causes	26 (4.6)	15(6.5)	11(3.3)	1.53(0.68-3.44)	0.2

*Of all subjects (n = 569). **Of never regular smokers (n = 232). ^+^Of ever regular smokers (n = 337). ^† ^Adjusted by age and gender.

## Discussion

We found an important statistically significant risk of CKD caused by smoking in hypertensive nephropathy and diabetic nephropathy patients and a weak, statistically insignificant association between smoking and CKD caused by glomerulonephritis.

Many studies indicate that the deleterious effect of smoking on renal function is not merely restricted to essential hypertension and diabetic nephropathy. Some of these studies found that smoking is an independent predictor of microalbuminuria in healthy patients with primary hypertension. It is well known that urinary albumin is a sensitive marker of glomerular injury [4], and the fact that there is a relationship between smoking and albuminuria indicates direct or indirect renal damage induced by smoking. Mimran *et al. *[17] studied lean subjects with essential hypertension and found that the prevalence of microalbuminuria was almost double in smokers as compared to non-smokers. Same results were found in other studies [18,19]. Multiple Risk Factor Intervention Trial (MRFIT) found an increased risk for end-stage renal disease (ESRD) for smokers as compared to non-smokers [20,21]. Such increased relative risk of ESRD related to smoking (up to 1.69 for heavy smokers) was independent of age, ethnicity, income, blood pressure, diabetes mellitus, prior history of myocardial infarction, or serum cholesterol.

Smoking causes kidney deterioration in diabetic patients with adverse effects on four different aspects of albumin excretion: It increases the risk of microalbuminuria[5], shortens the time interval between onset of diabetes and onset of albuminuria or proteinuria [6,7], accelerates the rate of progression from microalbuminuria to persistent proteinuria [22,23], and accelerates the rate of progression of diabetic nephropathy to ESRD [6,8]. Chase *et al. *[5] reported that in a group of 359 young subjects with IDDM the prevalence of borderline (> 7.6 mcg/min) and abnormal (> 30 mcg/min) albumin excretion rate was 2.8 fold higher in smokers than in non-smokers. Sawicki *et al. *[24] calculated the adjusted odds ratio for the progression of diabetic nephropathy and found that the odds ratio was higher by a factor of 2.74 for each 10 pack/year smoking history. In this study all patients were administered an intensified insulin along with antihypertensive therapy so that the confounding effects of hyperglycemia and hypertension were minimized. Similar results were found by Biesenbach *et al. *[8] which concluded that the rate of loss of GFR was higher by a factor of 1.44 in smoking patients as compared to the non-smoking patients with insulin dependent diabetes mellitus (IDDM), and by a factor of 1.66 among NIDDM patients.

To determine the risk factors for noninsulin dependent diabetes in a cohort representative of middle aged British men, Perry *et al. *[25] conducted a prospective study on 7,735 males (40 to 59 years) and found that smoking was associated with a 50% increased risk of developing NIDDM. This increased risk may be related to the fact that smoking aggravates insulin resistance in healthy smokers [26].

Another question would be the effect of smoking cessation on kidney function. In patients with IDDM, Chase *et al. *[5] found that albuminuria improved significantly when patients stopped smoking. Sawicki *et al. *[24] studied 34 smokers, 35 nonsmokers, and 24 ex-smokers with type I diabetes, hypertension, and diabetic nephropathy for one year and found that the progression of nephropathy was less common in nonsmokers (11%) than in smokers (53%) and in patients who had quit smoking (33%). They also found that accumulated dose measured by pack/year was an independent predictive factor for the progression of diabetic nephropathy.

One of the other diseases that exacerbate with smoking is autosomal dominant polycystic kidney disease (ADPKD), Chapman *et al. *[27] found that ADPKD patients with established proteinuria had a greater pack/year smoking history than their nonproteinuric counterparts did. Smoking is also responsible for renal function deterioration in lupus nephritis, as a retrospective cohort study of 160 adults found that smoking at the time of onset of nephritis was an independent risk factor for the more rapid development of ESRD [10]. The median time interval to terminal renal failure was 145 months in smokers, but more than 273 months in non-smokers. These effects persisted in multivariable analyses adjusting for differences among patients in age, gender, socioeconomic status, renal histology, and immunosuppressive treatment. They also found that the combination of smoking and hypertension resulted in shorter times to renal failure compared with nonsmoking hypertensive patients. This observation was not found in another study conducted on 70 lupus nephritis patients [28].

Appel *et al. *[29] studied 45 patients each 50 years of age or older, beginning renal replacement therapy and found that smoking aggravates renal artery stenosis. This effect was explained by increased arthrogenesis among smokers. In their study Wang *et al. *[30] stated that there is different susceptibility to arthrogenesis among smokers, and that the risk appears to be excessively high in patients who are homozygous for the endothelial nitric oxide synthase 4a (ecNOS4a) gene, which predisposes to endothelial dysfunction.

A previous, hospital-based case-control study [12] found a threefold increased risk for glomerulonephritis associated with CKD among heavy smokers while other studies did not find the same results. In a case-control study including 55 patients and 55 normal subjects Yaqoob *et al. *[31] reported that there was no significant correlation between tobacco and the development of primary glomerulonephritis. Merkel *et al. *[32] did not find any evidence that smoking aggravates the course of anti-glomerular basement membrane (anti-GBM) glomerulonephritis. Our study supports the observation made by Yaqoob regarding the association between smoking and the development of primary GN. However it was found that smoking is significantly associated with pulmonary hemorrhage in anti-GBM glomerulonephritis patients [33].

The increment of GFR induced by smoking may play a role in the genesis of hyperfiltration as a potential mediator of accelerated progression of chronic renal disease [34,35]. Pawlik *et al. *[36] studied renal excretory and circulatory responses to nicotine in anesthetized dogs and found that nicotine causes an increase in sodium and chloride excretion and urine flow, which is reversed after the administration of propranolol or in the group underwent adrenalectomy. This observation leads to a conclusion that nicotine action on the kidney is mediated by release of catecholamines from the adrenal medulla. Another mechanism could be the increment of vasopressin release secondary to altering cervical parasympathetic tone induced by nicotine, which increases GFR inappropriately [37,38]. On the other hand, the increment of GFR is thought to be due to an intermittent and persistent increase in blood pressure induced by smoking nicotine products; this blood pressure alteration was absent in patient smoking nicotine-free cigarettes [39-41].

Smoking also alters the proximal tubular function leading to increased excretion of N-acetyl-f3-glucosaminidase (NAG) and impairment of organic cation transport [42] which correlates strongly with the amount of tobacco consumed [13].

## Conclusions

In conclusion, our study found that smoking, particularly heavy smoking (> 30 pack/year), is an important risk factor to the development of CKD. The association was strongest for CKD classified as hypertension and diabetic nephropathy. These results raise the importance of smoking cessation to decrease the incidence of CKD and other preventable diseases as COPD, coronary artery diseases, and cancers.

## Competing interests

The authors declare that they have no competing interests.

## Authors' contributions

RY is the principle investigator, participated is study design, data analysis, data collection and manuscript preparation. HH participated on study design, data collection and manuscript preparation. AL participated in study design and manuscript preparation. RAA participated in data analysis and manuscript preparation. LV participated in data analysis and manuscript preparation. GA participated in data collection and manuscript preparation. NKA participated in data collection and manuscript preparation. SA participated in data collection. SAA participated in data collection. SAB is the supervisor, participated in study design and manuscript preparation.

All authors read and approved the final manuscript.

## Pre-publication history

The pre-publication history for this paper can be accessed here:

http://www.biomedcentral.com/1471-2458/10/731/prepub
